# Supplementary material for: Combined impacts of deforestation and wildlife trade on tropical biodiversity are severely underestimated
Source: Nat Commun. 2018 Oct 3;9:4052. doi: 10.1038/s41467-018-06579-2 (PMC6170487; doi:10.1038/s41467-018-06579-2)
Supplement: Supplementary file 1 — Supplementary Information [file 41467_2018_6579_MOESM1_ESM.pdf]

Supplementary information

Combined impacts of deforestation and wildlife trade on tropical biodiversity are severely underestimated

W.S. Symes[1,*], D. P. Edwards[2], J. Miettinen[3], F. E. Rheindt[1], L.R. Carrasco[1]

[1]Department of Biological Sciences, National University of Singapore, 14 Science Drive 4, Singapore 117543, Singapore.

[2]Department of Animal and Plant Sciences, University of Sheffield, Sheffield, S10 2TN, UK.

[3]Centre for Remote Imaging, Sensing and Processing (CRISP), National University of Singapore (NUS), 10 Lower Kent Ridge Road, Singapore 119076, Singapore.

[*]Email: wsymes@u.nus.edu. Tel: +6591377291. Fax: +65 67792486.

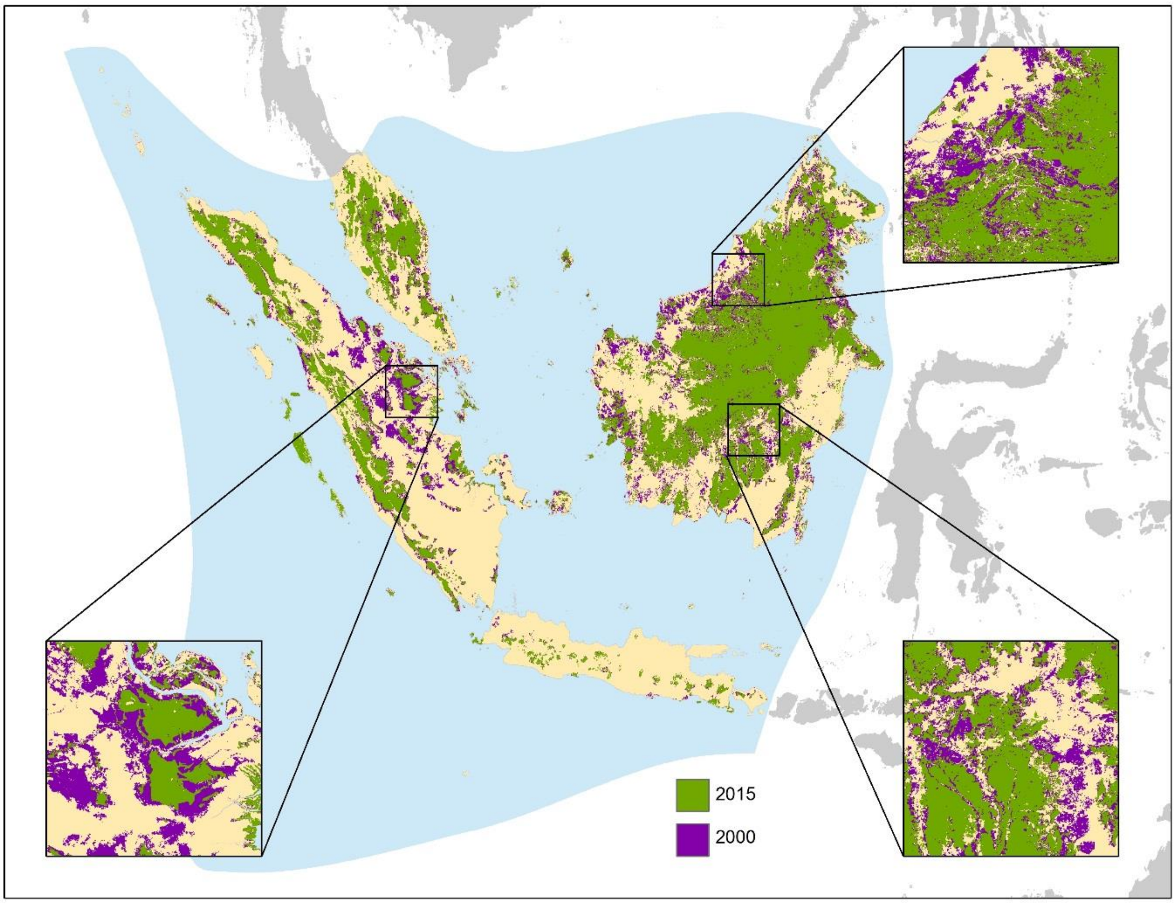

Supplementary Figure 1: Map of forest extent in Sundaland in 2000 (purple and green) and 2015 (green). The blue area is the extent of Sundaland. The three insets are highlighting forest loss in (clockwise from the top right) northern Sarawak and central Kalimantan in Borneo and Riau in Sumatra. © EuroGeographics for the administrative boundaries

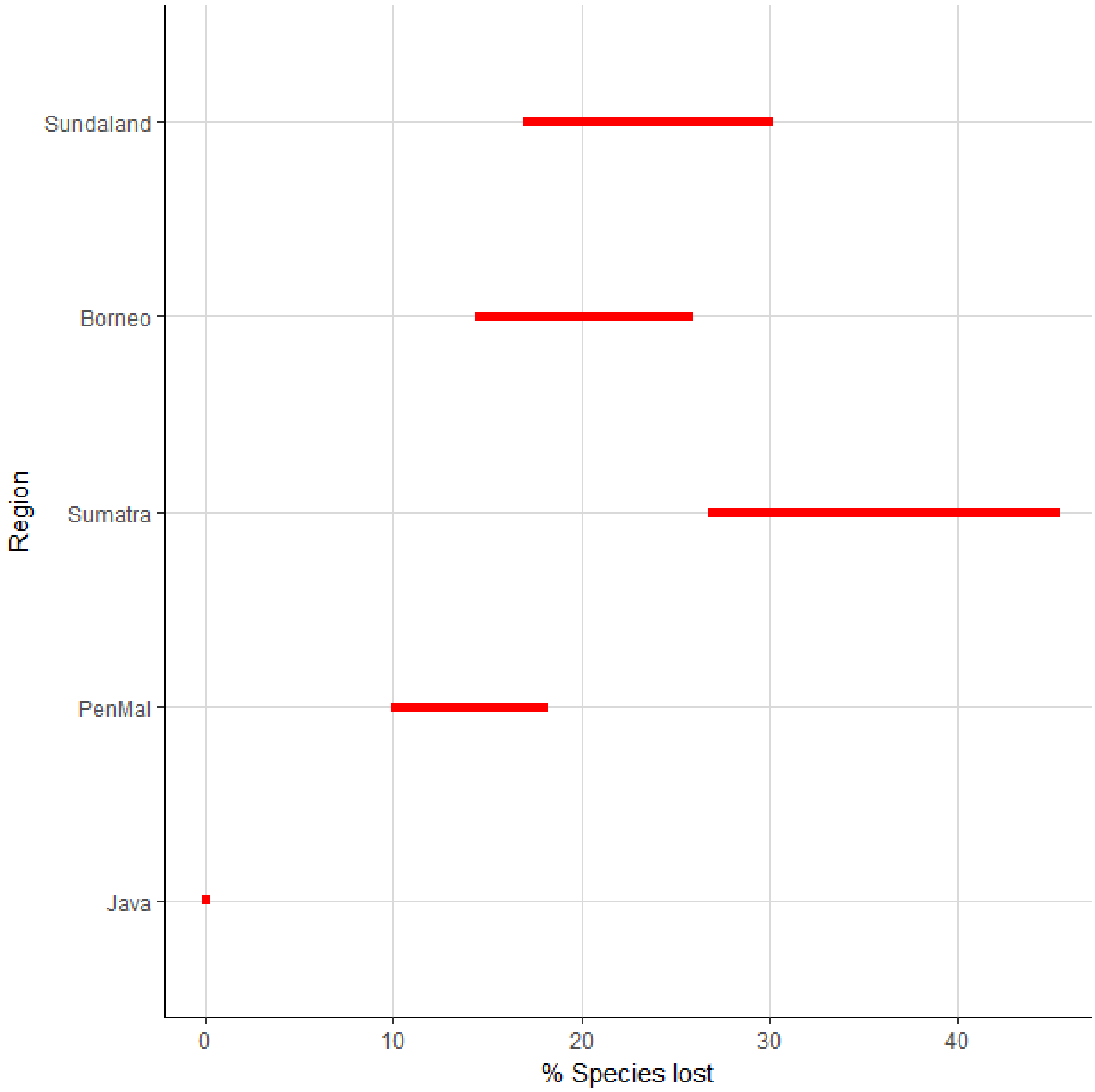

Supplementary Figure 2: Percentage of species expected to be lost on each Island and in the region by 2100 at current rates of deforestation, according to the species area relationship. The upper value represent a z value of 0.35 and the lower end 0.21. PenMal = Peninsular Malaysia.

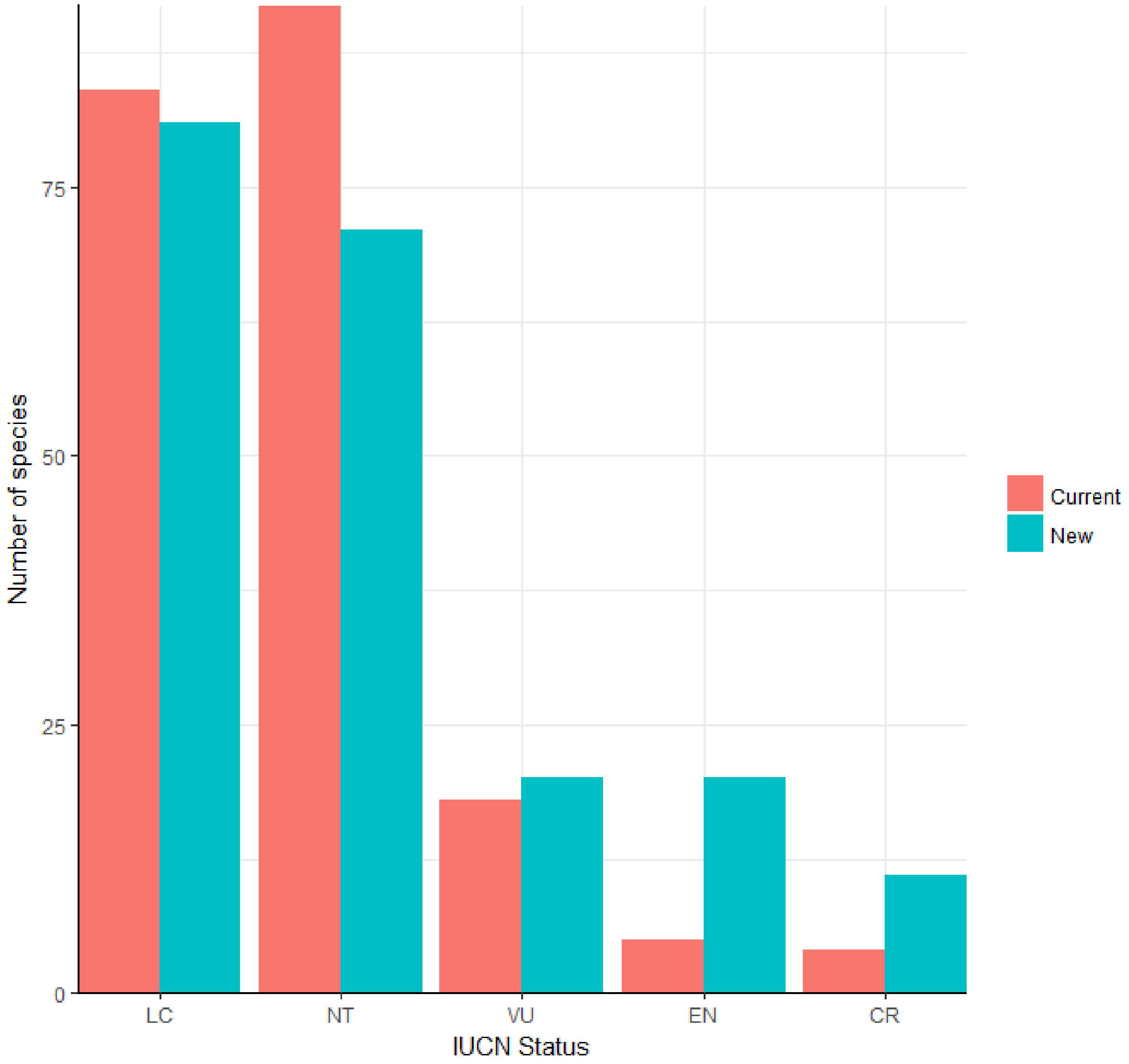

Supplementary Figure 3: Bar chart comparing the current IUCN status and the new status based on our analysis of the 203 Sundaland forest dependent birds in our analysis.

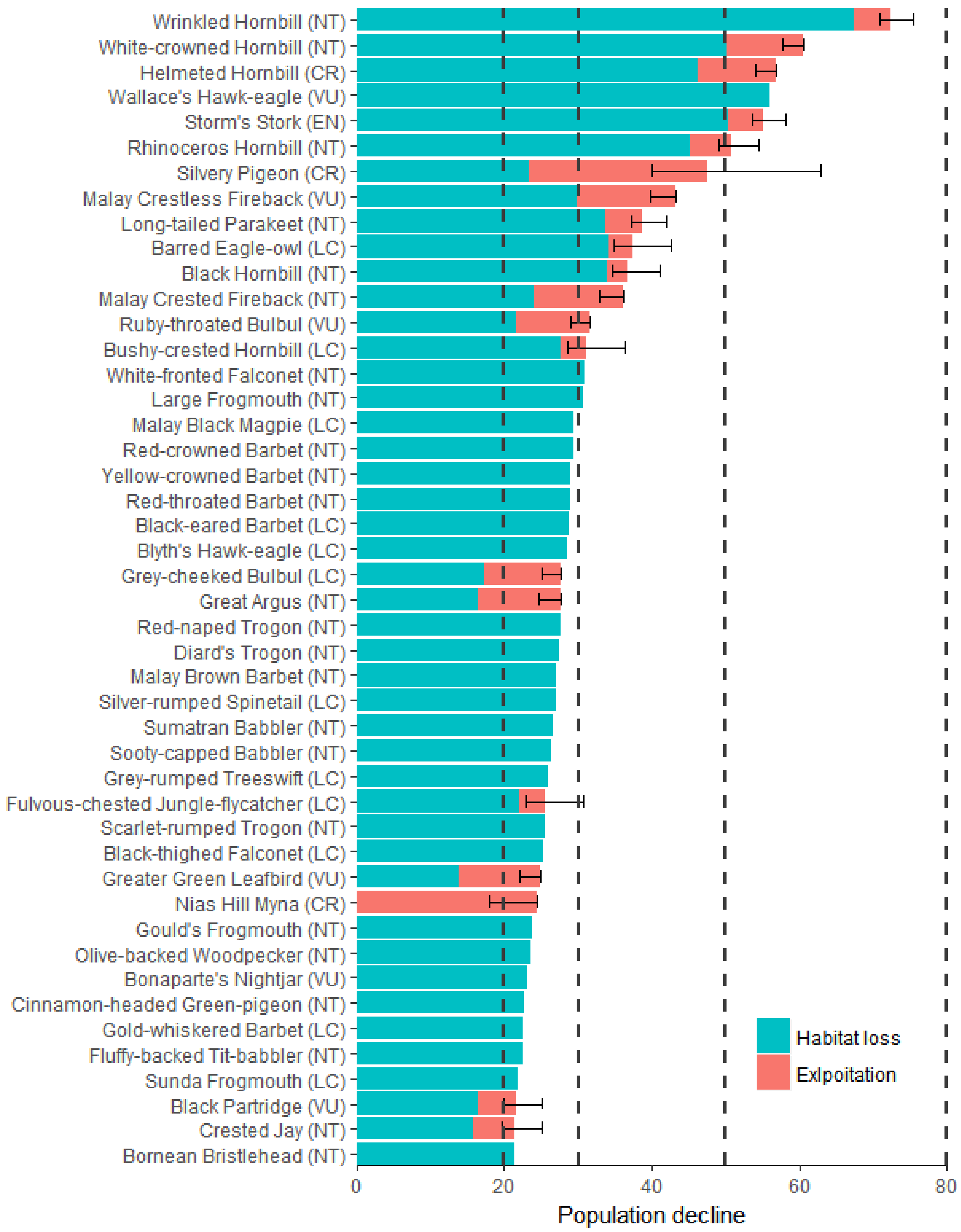

Supplementary Figure 4: Combined population declines from habitat loss and trapping using only distance to major roads. The blue bar is the contribution of habitat loss and the red bar the contribution of trapping. This graph only shows the 45 regionally endemic species with the highest predicted declie (for all species see SOM). Error bars represent the uncertainty in our estimates of population decline due to trapping. Vertical lines represent the thresholds for classification as Near Threatened (NT) (20%), Vulnerable (VU) (30%), Endangered (EN) (50%) and Critically Endangered (CR) (80%).

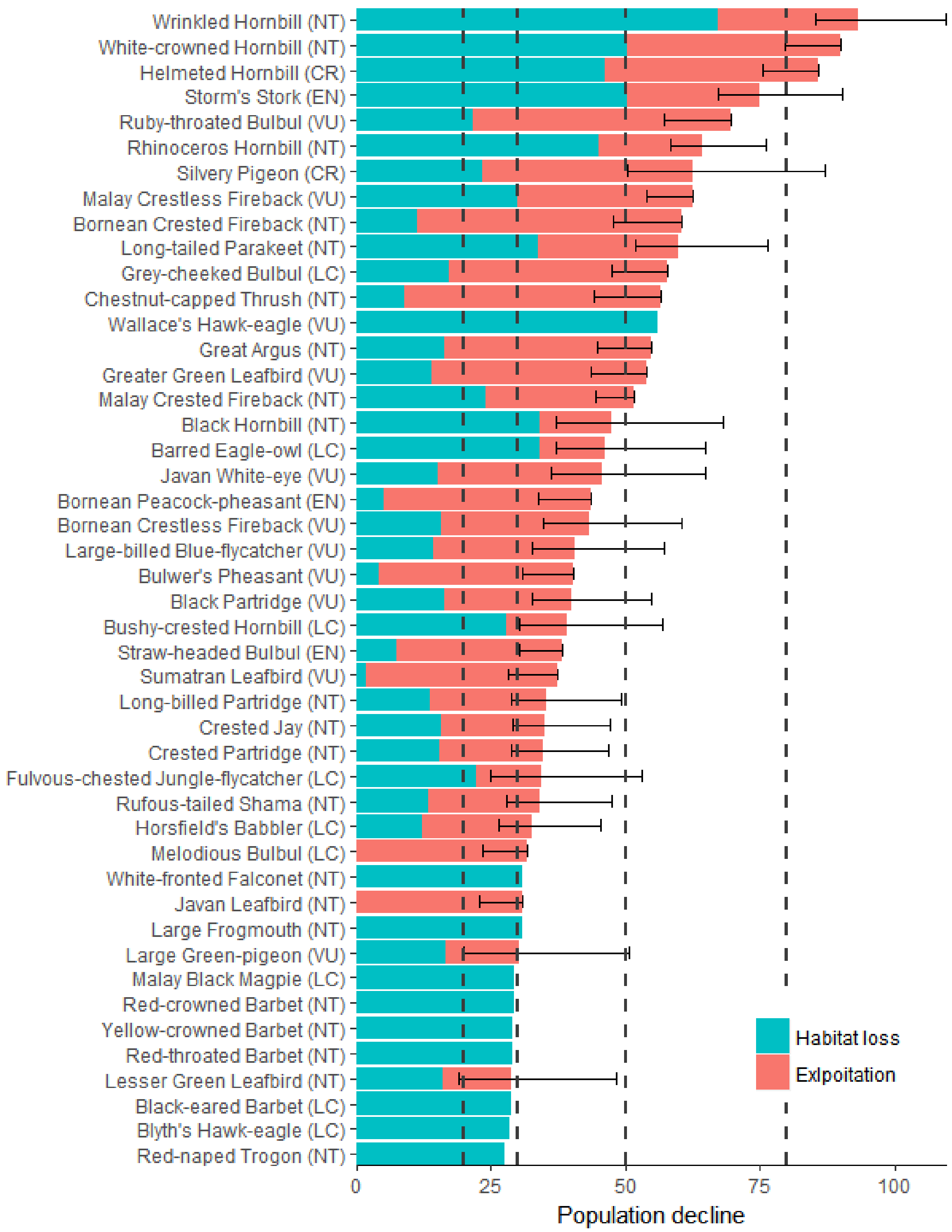

Supplementary Figure 5: Combined population declines from habitat loss and trapping using distance to all roads. The blue bar is the contribution of habitat loss and the red bar the contribution of trapping. This graph only shows the 45 regionally endemic species with the highest predicted decline (for all species see SOM). Error bars represent the uncertainty in our estimates of population decline due to trapping. Vertical lines represent the thresholds for classification as Near Threatened (NT) (20%), Vulnerable (VU) (30%), Endangered (EN) (50%) and Critically Endangered (CR) (80%).
