# Supplementary material for: Combined impacts of deforestation and wildlife trade on tropical biodiversity are severely underestimated
Source: Nat Commun. 2018 Oct 3;9:4052. doi: 10.1038/s41467-018-06579-2 (PMC6170487; doi:10.1038/s41467-018-06579-2)
Supplement: Supplementary file 3 — Description of Additional Supplementary Files [file 41467_2018_6579_MOESM3_ESM.pdf]

Legends for Supplementary Data 1 and Supplementary Data 2 files:

Supplementary Data 1: Summary of the results for regionally endemic species (defined as having greater than 80% of their range inside Sundaland), the columns are as follows: Scientific name, Common Name, Persecution, Persc_source (LH = local hunting, DM = domestic market, IT = international trade), Area_2015km2 (the Extent of Suitable Habitat in 2015), Perc Change 00-15 (Percentage change in ESH between 2000 and 2015), Gen time (Generation time), expected hloss 3 gen (the expected habitat loss in 3 generations or 10 years), hunting impact, perc sundaland (percentage of ESH in Sundaland), Combined hunting hloss (the combined population decline due to hunting and habitat loss), per PA (percentage of ESH inside a protected area), PA area15 km2 (the total ESH inside PAs in 2015), Current Red List (current red list status), Hloss Red List (the Red List status based only on the rate of habitat loss), Hunt Red List (Red list status based only on declines from hunting), Combined Red List (Red List status based on habitat loss and hunting), notes.

Supplementary Data 2: Summary of the results for non-regionally endemic species (defined as having less than 80% of their range inside Sundaland), note the Red List categories are for the regionally status of the species and not recommendation for status changes, the columns are as follows: Scientific name, Common Name, Persecution, Area_2015km2 (the Extent of Suitable Habitat in 2015), Perc Change 00-15 (Percentage change in ESH between 2000 and 2015), Gen time (Generation time), expected hloss 3 gen (the expected habitat loss in 3 generations or 10 years), hunting impact, perc sundaland (percentage of AOO in Sundaland), Combined hunting hloss (the combined population decline due to hunting and habitat loss), per PA (percentage of ESH inside a protected area), PA area15 km2 (the total ESH inside PAs in 2015), Current Red List (current red list status), Hloss Red List (the Red List status based only on the rate of habitat loss), Hunt Red List (Red list status based only on declines from hunting), Combined Red List (Red List status based on habitat loss and hunting).
